# Mepolizumab-Induced Posterior Reversible Encephalopathy Syndrome (PRES), a new patient report

**DOI:** 10.1186/s12883-022-02849-1

**Published:** 2022-08-25

**Authors:** Vikram V. Puram, Dana Ghazaleh, Apameh Salari, Kaci McCleary, Gerald Moriarty, Kendall Nichols, Malik Ghannam, Kevin Brown, Brent Berry

**Affiliations:** 1 University of Minnesota Medical School, 420 Delaware St SE, Minneapolis, MN 55455 USA; 2 Department of Neurology, University of Iowa Hospitals and Clinics, 200 Hawkins Dr, Iowa City, IA 52242 USA; 3 Department of Neurology, University of Minnesota, 516 Delaware St SE, Minneapolis, MN 55455 USA; 4 VA Medical Center, 1 Veterans Drive, Minneapolis, MN 55417 USA; 5 Dept. of Neurology, Dept. of Sleep Medicine, Dept. of Physiology and Biomedical Engineering, Mayo Clinic, 200 First St. SW, Rochester, MN 55905 USA; 6 BioTechMed Center, Department of Multimedia Systems, Faculty of Electronics, Telecommunication and Informatics, Gdansk University of Technology, 8 Jana Bażyńskiego, 80-309 Gdańsk, Pomeranian Voivodeship Poland

**Keywords:** Mepolizumab, Posterior reversible encephalopathy syndrome, Asthma, Drug reaction

## Abstract

**Background:**

Posterior Reversible Encephalopathy Syndrome (PRES) is a neurotoxic state characterized by seizures, headache, vision change, paresis, and altered mental status. PRES has an important place in medicine due to the wide variety of causative diseases, infections, and medications that precipitate its mysterious onset. Although exposure to medications, particularly immunosuppressants, cancer chemotherapy, and biologic drugs, is a common occurrence in patients who develop PRES, Mepolizumab has never before been associated.

**Case presentation:**

This report of a 67-year-old male patient outlines the first reported case of Mepolizumab-induced PRES in the literature.

**Conclusions:**

Treatment of severe asthma, asthma-exacerbations, and diseases such as eosinophilic granulomatosis with polyangiitis (formerly Churg-Strauss) with Mepolizumab is rapidly gaining popularity ever since the drug’s recent FDA-approval. This report aims to raise awareness of this potentially life-threatening and previously unreported side effect of Mepolizumab since early identification of the causative agent is the key to preventing the severe neurologic disability and possible death that may occur from the delayed treatment of PRES.

## Background

Mepolizumab (NUCALA) is a humanized monoclonal antibody antagonist to IL-5, recently approved by the FDA in 2015. It has gained popularity in the treatment of severe eosinophilic asthma and eosinophilic granulomatosis with polyangiitis (Churg-Strauss syndrome). This report is the first in the literature to identify the IL-5 directed antibody, Mepolizumab, as a potential causative agent of Posterior Reversible Encephalopathy Syndrome (PRES) also known as Reversible Posterior Leukoencephalopathy [1–3]. PRES is a clinico-radiologic syndrome of heterogeneous etiologies that is diagnosed upon both identification of specific clinical manifestations indicative of a neurotoxic state as well as pathognomonic radiologic criteria such as subcortical brain edema.

## Case presentation

A 67-year-old male presented to the emergency department with an acute event of altered awareness. The patient was watching TV with his family when he suddenly had an unusual sensation of sand in his eyes. His family noticed that he was verbally unresponsive for 15 min with a blank stare. There were no abnormal movements of the body or limbs. He scratched his head and stood up during the episode but did not remember this. The patient maintained postural tone and there was no loss of bowel or bladder control. When he regained speech, it was slurred and incoherent. He presented to our emergency department by ambulance and was back to baseline when he was evaluated with no apparent recollection of the event.

He endorsed 4 weeks of new-onset worsening bifrontal headaches and new-onset episodes of pins and needles in his extremities. Prior to that, he reported a normal state of health. He denied fevers, chills, night sweats, stiff neck, or weight change. The patient has a medical history of chronic asthmatic bronchitis, obstructive sleep apnea, hypertension, and well-controlled type II diabetes on daily metformin. His asthma was well controlled on Omalizumab, but two months prior, he changed to Mepolizumab due to personal preference for a lower dosing frequency. The patient had been newly prescribed 100 mg/mL of Mepolizumab on a dosing frequency of every 4 weeks and it had been taken twice prior to his hospital admission (last dose two weeks prior). His eosinophil counts (absolute) were 170, 60, 70, and 310 / uL at his prior outpatient visits over the previous 18 months.

On examination by neurology, the patient was fully alert, attentive, and oriented. He provided a coherent history with no difficulty answering questions or following commands. Speech was slow and mildly dysarthric, but the patient and his wife state that this was his baseline. Language was fluent without paraphasic errors. Naming and repetition were intact. No extinction or neglect was elicited. His left pupil was irregular and larger than the right (noted to have prior cataract surgery in that eye). Both were briskly reactive. Visual fields were intact. His extraocular movements showed saccadic breakdown of pursuit, and he had multiple beats of bidirectional end-gaze nystagmus with intermittent rotary qualities. His facial sensation was symmetrically intact. He had unilateral flattening of the nasolabial fold which corrected with a smile (patient stated this was normal). Shoulder shrug was strong bilaterally. Tongue protrusion was midline with full rapid lateral motion. No asterixis, tremors or other abnormal movements were observed. He had a normal muscular tone throughout and 5/5 bilateral upper and lower extremities without pronator drift. Biceps and brachioradialis reflexes were 1 + and symmetric. Patellar and ankle jerk reflexes were 2 + and symmetric. Hoffman’s and Babinski’s signs were not noted and clonus was absent. His sensation to light touch and pinprick was intact throughout. He reported decreased vibratory sensation at left medial malleolus compared to right, although both were near normal. Dysmetria was present on both bilateral finger-nose-finger and heel-shin testing. Rapid alternating movements were intact. He took a few steps with a normal and stable, casual gait.

**Fig. 1:**
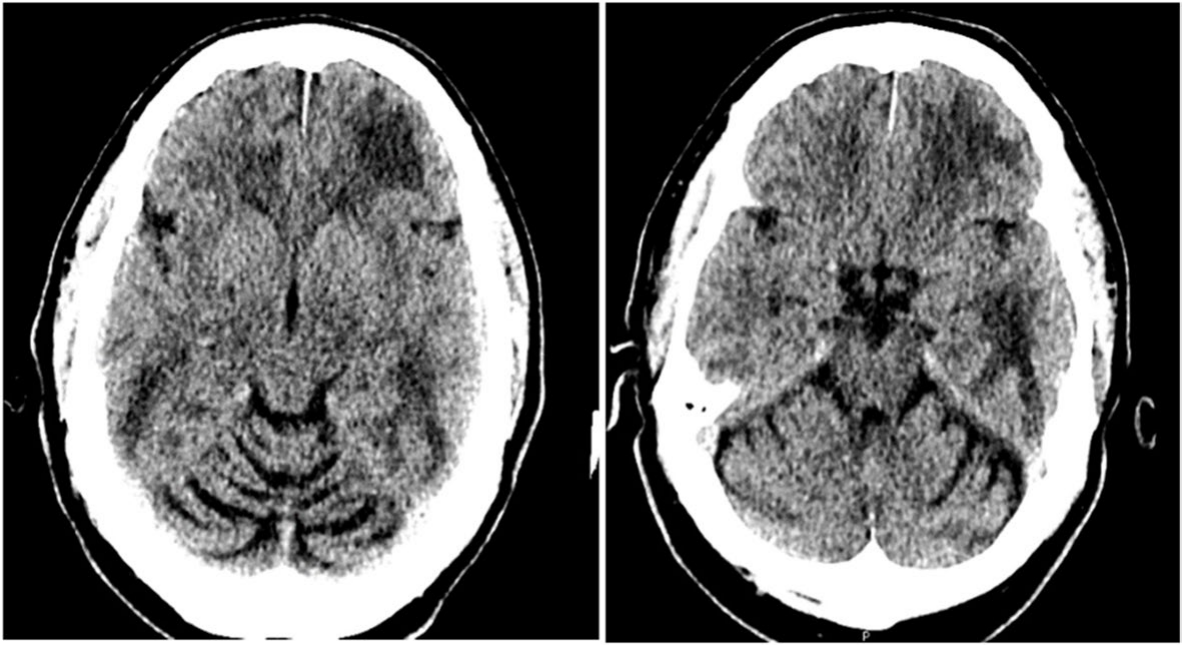
CT without contrast, initial scan on presentation. This head CT revealed confluent areas of white matter hypoattenuation bilaterally, slightly worse on the left, involving the subcortical white matter. No significant mass effect midline shift, or hemorrhage. The differential was thought to include leukoencephalopathy, vasculitis, or infiltrating neoplasm such as lymphoma among other etiologies

The patient was admitted to the hospital for observation and workup of his isolated episode of altered mental status as well as his new headaches and paresthesias. He was started on Keppra (levetiracetam) for possible complex-partial seizure. The initial differential included hypetensive encephalopathy, cerebral venous thrombosis, acute demyelinating encephalomyelitis, malignancy (primary or metastatic) or paraneoplastic encephalitis, infectious encephalitis, and various toxidromes (such as lead, carbon monoxide, inhalants, amphetamines, opiates, and/or benzodiazepines). Head CT revealed bilateral areas of confluent subcortical white matter hypoattenuation (Fig. 1). The differential was narrowed to include leukoencephalopathy, vasculitis, or infiltrating neoplasm such as lymphoma among other etiologies.

**Fig. 2:**
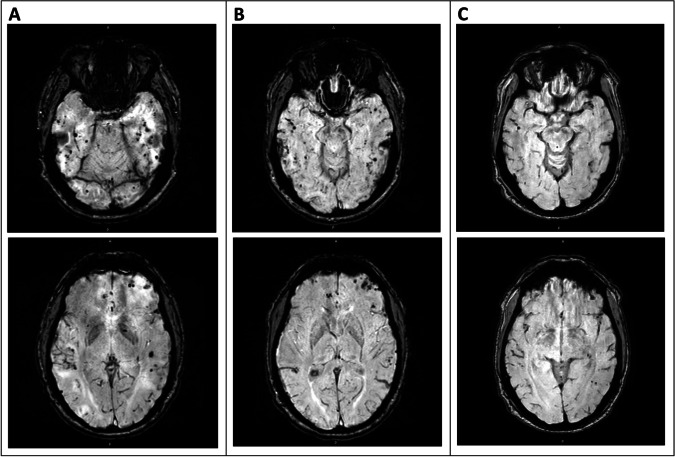
SWI MRI sequences both high and low cuts (**A**) Initial Presentation (**B**) 1-month after stopping Mepolizumab **C**) 6-months after stopping Mepolizumab

He had an EEG which showed left frontal sharps and spikes with focal slowing and mild generalized slowing. MRI was conducted 35 h after initial CT scan with findings characteristic for PRES (Fig. 2). Labs, including lumbar puncture (glucose 69, protein 78, RBC 4, WBC 0) were unremarkable. His blood pressure had been stable in the 130–160/70–90 mmHg range over the previous 12 months and the patient had no recent changes to his regimen (candesartan). The patient’s blood pressure values during two unrelated hospital visits within the two months prior to hospitalization were 137/76 mmHg (MAP 96) and 129/71 mmHg (MAP 90). During the hospitalization, the patient’s blood pressure ranged between 137/68 mmHg (MAP 91) and 148/78 mmHg (MAP 102) with a mean MAP of 94 across the hospitalization. His other vitals were also unremarkable.

**Fig. 3:**
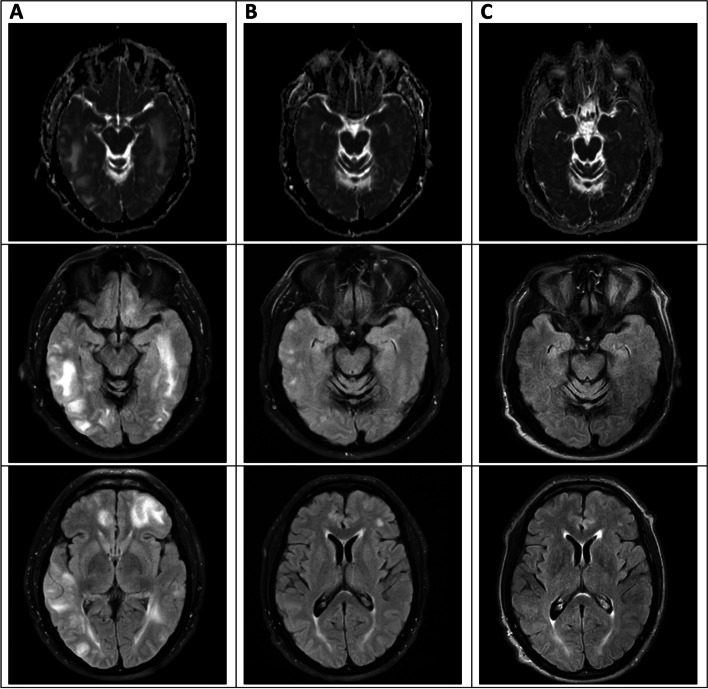
MRI sequences related to patient’s PRES. Top row Diffusion, Middle and Bottom Row FLAIR (**A**) Initial Presentation (**B**) 1-month after stopping Mepolizumab (**C**) 6-months after stopping Mepolizumab. Underlying white matter edema. There is also an unusual pattern of associated susceptibility artifact, which might imply a process that is hemorrhagic. In all sequences assessed, this patient’s scan is notably improved

PRES was ultimately identified due to the suspicious temporal relationship between Mepolizumab initiation and the onset of symptoms in conjunction with pathognomonic imaging findings. Though other IL-modulating drugs such as tocilizumab and ustekinumab have been implicated in PRES, there are no previous reports of Mepolizumab-associated PRES; this suspicion was relayed to the patient’s pulmonology team, however, due to lack of evidence and worsening control of the patient’s respiratory symptoms, Mepolizumab was re-ordered. The patient ultimately declined re-starting the drug due to the recommendations from neurology. Symptoms and imaging showed improvement at both 1-month and 6-month follow up appointments (Figs. 2 and 3). Due to the absence of seizures or any new episode of altered awareness, the patient was titrated off his levetiracetam dose and has not reported any more issues or concerns. He has transitioned back to omalizumab and has regained good control of his asthma.

## Discussion

PRES was first described in a case series in 1996 by Hinchey et. al. in the New England Journal of Medicine where four syndromes (Reversible Posterior Cerebral Edema Syndrome, Posterior Leukoencephalopathy Syndrome, Hyperperfusion Encephalopathy, Brain Capillary Leak Syndrome) were codified under a single name [4]. Unfortunately, none of these names are satisfactory as the syndrome is, confusingly, not always reversible and it is not always confined to the white matter nor the posterior regions of the brain [5]. However, it is postulated that the posterior brain is more commonly involved due to the slightly lower concentrations of autoregulatory adrenergic nerves around the pial and intracerebral vessels posteriorly compared to anterior circulation [6]. PRES affects women more than men and is seen most often in age 40–50 [4].

The pathophysiology of PRES is unclear, although two primary theories have been postulated [7]. The hyperperfusion theory proposes that hypertension, or even simply a rapid increase from the patient’s baseline blood pressure disrupts basement membrane integrity in the blood–brain barrier (BBB), causing extravasation of fluid and subsequent vasogenic edema (VE). Consistent with this theory, patients with chronic hypertension are at increased risk of developing PRES, and acute increases in blood pressure are present in 60–80% of PRES patients [7]. Conversely, the hypoperfusion theory proposes that cell cytotoxicity through mechanisms such as autoimmune disease, immunosuppression, and cancer chemotherapy can cause similar BBB disruption resulting in VE. PRES has been described extensively in the use of immunosuppressant drugs such as gemcitabine, tacrolimus, and sirolimus. Commonly used biologics such as Adalimumab (Humira), Infliximab (Remicade), Rituximab (Rituxan), and Etanercept (Enbrel) have all been implicated in numerous PRES cases as well. The high percentage of patients with PRES who have autoimmune disorders may further support the theory that PRES is in part caused by endothelial dysfunction, a process in which the host autoimmune response is essential; endothelial cells become activated and damaged by an inflammatory cytokine response stemming from monocytes and lymphocytes, which can lead to leakage of fluid and protein into the interstitium [8–11].

The symptoms of PRES evolve rapidly over hours to days and include seizures, headaches, altered consciousness, and visual disturbances often in the context of elevated BP as discussed above. Seizures are often the presenting symptom with only a small minority of patients with mild disease being seizure-free. Though noncontrast head CTs are often the first neuroimaging study performed in the emergency department, follow-up neuroimaging with noncontrast brain MRI is essential to an accurate diagnosis. Elimination of other differential diagnoses such as hypertensive encephalopathy and reversible cerebral vasoconstriction syndrome is critical as PRES can mimic many other disease processes. It is necessary to treat PRES promptly to prevent permanent neurological damage or even death. Treatment typically consists of antiseizure medications and removal of the offending agent along with strict blood pressure control.

## Conclusions

Reversible posterior leukoencephalopathy syndrome (PRES) is an increasingly recognized neurological disorder with an ever-increasing number of documented etiologies. It most often presents with clinical seizures, headache, and visual symptoms, all three of which were seen in this case report. Seizures have been noted to appear slightly later in onset relative to headaches, consistent with the 4-weeks of new-onset headaches leading up to our patient’s episode of altered mental status and subsequent 911 call. The relationship between PRES and mepolizumab remains unknown however this case report is the first of its kind. It hopes to highlight the importance for prescribers of mepolizumab such as pulmonologists, rheumatologists, and immunologists as well as physicians who care for patients on mepolizumab to identify signs and symptoms so that early discontinuation of the drug can prevent the permanent damage and even death that may occur from delayed treatment of PRES.

## Data Availability

All the data supporting our findings is contained within the manuscript. Data sharing is not applicable to this article as no datasets were generated or analysed during the current study.

## Abbreviations

PRES: Posterior Reversible Encephalopathy Syndrome
BBB: Blood–Brain Barrier
VE: Vasogenic Edema
